# Causal relationship between plasma metabolites and carpal tunnel syndrome risk: evidence from a mendelian randomization study

**DOI:** 10.3389/fmolb.2024.1431329

**Published:** 2024-10-03

**Authors:** Wenbao Wu, Daofeng Fan, Chong Zheng, Binfu Que, Qing qing Lian, Yangui Chen, Rui Qiu

**Affiliations:** ^1^ Department of Acupuncture and Moxibustion, Longyan First Hospital Affiliated to Fujian Medical University, Longyan, China; ^2^ Department of Neurology, Longyan First Hospital Affiliated to Fujian Medical University, Longyan, China

**Keywords:** carpal tunnel syndrome, mendelian randomization, metabolites, glucuronate, cysteinylglycine disulfide, adenosine 5′-monophosphate to phosphate ratio

## Abstract

**Background:**

Carpal tunnel syndrome (CTS) is a common symptom of nerve compression and a leading cause of pain and hand dysfunction. However, the underlying biological mechanisms are not fully understood. The aim of this study was to reveal the causal effect of circulating metabolites on susceptibility to CTS.

**Methods:**

We employed various Mendelian randomization (MR) methods, including Inverse Variance Weighted, MR-Egger, Weighted Median, Simple Mode, and Weighted Model, to examine the association between 1,400 metabolites and the risk of developing CTS. We obtained Single-nucleotide polymorphisms (SNPs) associated with 1,400 metabolites from the Canadian Longitudinal Study on Aging (CLSA) cohort. CTS data was derived from the FinnGen consortium, which included 11,208 cases and 1,95,047 controls of European ancestry.

**Results:**

The results of the two-sample MR study indicated an association between 77 metabolites (metabolite ratios) and CTS. After false discovery rate (FDR) correction, a strong causal association between glucuronate levels (odd ratio (OR) [95% CI]: 0.98 [0.97–0.99], p _FDR_ = 0.002), adenosine 5′-monophosphate (AMP) to phosphate ratio (OR [95% CI]:0.58 [0.45–0.74], p _FDR_ = 0.009), cysteinylglycine disulfide levels (OR [95% CI]: 0.85 [0.78–0.92], p _FDR_ = 0.047) and CTS was finally identified.

**Conclusion:**

In summary, the results of this study suggest that the identified glucuronate, the ratio of AMP to phosphate, and cysteinylglycine disulfide levels can be considered as metabolic biomarkers for CTS screening and prevention in future clinical practice, as well as candidate molecules for future mechanism exploration and drug target selection.

## 1 Introduction

Carpal tunnel syndrome (CTS) emerges when the median nerve gets compressed within the carpal tunnel by the transverse carpal ligament (Carpal tunnel syndrome, 2019; Walter, 2022). This condition is primarily marked by symptoms such as paresthesia, numbness, and a “pins and needles” sensation in the thumb, index, and middle fingers (Newington et al., 2015). Some individuals may also experience muscle atrophy in the thenar area, difficulty with thumb abduction, and palm dysfunction (Middleton and Anakwe, 2014). CTS is notably prevalent, affecting 5%–10% of the population, particularly middle-aged and pregnant women (Atroshi et al., 1999). Although surgical decompression provides relief for many, a portion of patients endure persistent or recurring symptoms, making CTS a significant socioeconomic challenge. The incidence of CTS is on the rise (Hacquebord et al., 2022), leading to reduced hand function, decreased work efficiency, and increased medical costs. Consequently, finding effective prevention methods for CTS is a critical area of research.

Despite its common occurrence, the underlying pathophysiology of CTS, especially concerning its genetic aspects, remains poorly understood. Metabolomics, the study of metabolites in biological samples, offers promising insights into the altered metabolic pathways associated with diseases like CTS (González-Domínguez et al., 2020). By examining metabolite levels, researchers can discern variations in normal and pathological states (Teckchandani et al., 2021a). This approach can improve our understanding, diagnosis, and management of CTS. However, research specifically focusing on the application of metabolomics to CTS is still limited, highlighting a significant need for further study.

This research employed the MR method to explore the relationship between metabolomics and CTS. Clinical research often relies on observational studies and randomized controlled trials, with the latter being the gold standard. However, challenges such as ethical constraints and high costs can limit the feasibility of conducting randomized controlled trials. Observational studies, while simpler to implement, are often subject to confounding factors and reverse causality issues (Sekula et al., 2016). MR utilizes genetic variations as instrumental variables to examine causal relationships, thus bypassing these limitations (Bowden and Holmes, 2019). This method provides a more reliable inference of causality between disease and genetic factors. Our study applies MR to investigate the causal relationship between plasma metabolites and CTS, aiming to contribute to the management and prevention of this condition.

## 2 Methods

### 2.1 Study design

**FIGURE 1:**
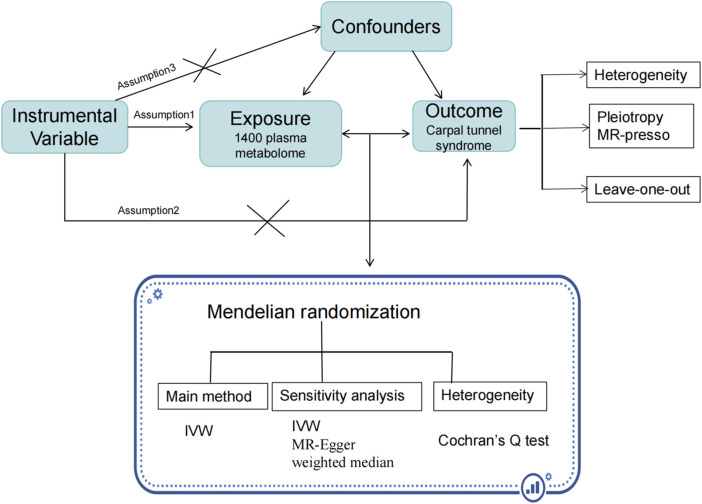
The study flow chart. Legend: Figure 1 illustrates the flow of participants through the study. This visual representation provides a clear overview of the study design and participant retention throughout the research process. X indicates that there is no correlation.

Figure 1 presents a comprehensive overview of our study design, underpinned by three key assumptions integral to the MR methodology. The first assumption posits a robust correlation between specific genetic variants and the exposure factor, in this case, metabolites related to CTS. The second assumption stipulates that the instrumental variables, derived from genetic variants, are not influenced by any external confounding factors. This ensures the observed effects are primarily due to the genetic variants themselves. The third assumption is that these instrumental variables are not directly linked to the outcomes, barring their effect through the exposure. In this study, our focus was on the relationship between plasma metabolites and CTS. To this end, we analyzed 1,400 metabolites, data which were sourced from the Canadian Longitudinal Study on Aging (CLSA) cohort and are available through the Genome-Wide Association Studies (GWAS) Catalog database (GWAS:GCST90199621-90201020). Information specific to CTS was obtained from the FinnGen database. The inclusion of these studies in GWAS was contingent upon approval from the respective review committees and the informed consent of all participants. The present study was approved by the ethics committee of Longyan First Hospital (Ethics number: 2,022,022).

### 2.2 Instrumental variable selection

We identified 34,930 single-nucleotide polymorphisms (SNPs) associated with 1,400 metabolites at the genome-wide significance level (*p*-value < 1 × 10^−6^). This identification was based on data from the he CLSA cohort (Chen et al., 2023). To ensure the independence of these instrumental variables (IVs) representing the 1,400 metabolites, we employed a stringent selection process. This process involved (1) clumping, an approach that involves grouping SNPs that are in high LD with each other to identify and retain only one representative SNP from each group, thereby reducing redundancy and ensuring that the selected SNPs are not correlated, and (2) discarding SNPS that exhibited a linkage disequilibrium (LD) R^2^ greater than 0.01 or were within a ±500 kilobase (kb) distance to ensure further that SNPs within this genomic distance of each other were not included simultaneously if they were in LD. This study applied these criteria to select a set of independent SNPs, ensuring that the genetic variation captured by each SNP is distinct and not influenced by the variation captured by other SNPs in the set. This independence is essential for the validity of our instrumental variable analysis, as it reduces the risk of bias and ensures that the associations we observe are more likely to be due to the causal effects of the genetic variants rather than confounding factors. To identify the effect of confounding factors on CTS, all SNPs were searched by using the ensembl database (https://www.ensembl.org/index.html). Through the analysis, it was found that some SNPs associated with each metabolite were also associated with a variety of CTS risk factors including diabetes mellitus, dyslipidemia, thyroid disease, overweight or obesity, etc. By controlling for confounding factors that may affect CTS outcomes, 7,373 SNPs can be identified to serve as instrumental variables (detailed in Supplementary Table S1). Furthermore, we calculated the F-statistics for these SNPs to evaluate the strength of the genetic variation they represent. The F-statistics for all selected SNPs were found to be greater than 10, indicating a strong and reliable genetic instrument for our MR analysis.

### 2.3 CTS data source

Summary-level data on the association of outcome-related SNPs with CTS was obtained from a genome-wide association meta-analysis conducted by the FinnGen consortium. The dataset included 11,208 CTS cases and 1,95,047 controls of European descent. The data can be accessed at https://www.finngen.fi/en.

### 2.4 Statistical analysis

To assess the relationships between 1,400 metabolites and CTS, our study employed a two-sample MR method. The primary statistical approach was the Inverse Variance Weighted (IVW) method. For cases where the IVs were three or fewer, we combined the Wald ratio of a SNP effect on the outcome using the fixed-effect IVW method. In instances with more than three IVs, the random-effect IVW method was utilized. We also used MR-Egger regression, weighted medians, and both a simple and weighted model for auxiliary analysis. The MR-Egger regression method was used to correct for horizontal pleiotropy (*p*-value for intercept <0.05), enhancing the credibility of MR analysis (Carter et al., 2021). We used the weighted median method, enhancing causal effect estimate accuracies by weighing genetic variation locus effects (Davies et al., 2018). We applied the simple mode test to determine the significance of genotype differences, providing insights into gene effects on specific traits (Birney, 2022). Utilizing a weighted model accounted for sample importance differences, enhancing result accuracy and reliability, which is especially pertinent when interpreting complex genetic data and exploring gene impacts on specific traits (Davey Smith and Hemani, 2014). Sensitivity analyses were conducted using the MR-Egger method and MR-PRESSO. The MR Steiger test was utilized to determine the causal direction between metabolites and CTS. A statistically significant association was considered if the estimated causal effect of a given metabolite had a FDR <0.05. The analyses were performed using the TwoSample MR package (version 0.5.7), MendelianRandomization (version 0.5.7) and MR-PRESSO (version 1.0) in R (version 4.3.1).

## 3 Results

**FIGURE 2:**
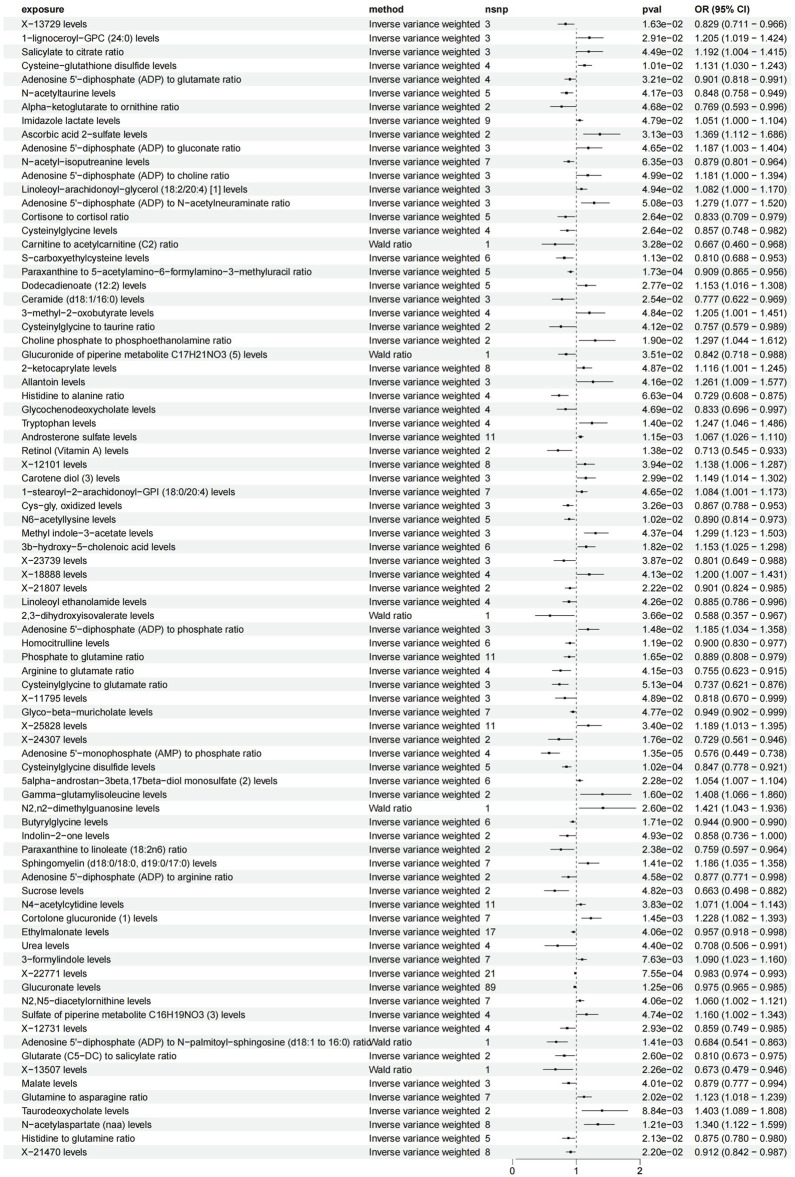
77 metabolites with IVW as the primary outcome forest plot. Legend: Figure 2 presents a forest plot displaying the results of the inverse variance weighted (IVW) analysis for 77 metabolites.

**FIGURE 3:**
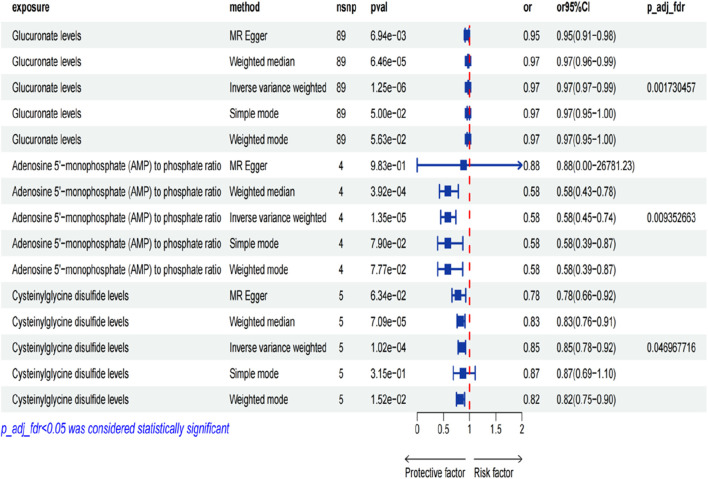
Plot of metabolites with significant presence after FDR correction. Legend: Figure 3 displays a scatter plot of metabolites with significant presence after false discovery rate (FDR) correction.

We investigated the influence of 1,400 metabolites on CTS, identifying significant causal relationships through the application of the Inverse Variance Weighted (IVW) method, as detailed in Supplementary Tables S2, S3. The findings from a two-sample MR study revealed an association between 77 metabolites (and metabolite ratios) and CTS, as illustrated in Figure 2 (detailed in Supplementary Table S4). There are 38 metabolites (metabolite ratios) that may be protective factors for CTS and another 36 that may be risk factors for CTS (as detailed in Supplementary Tables S5, S6). Following correction for the false discovery rate (FDR), a robust causal link was established between glucuronate levels, the ratio of AMP to phosphate, and cysteinylglycine disulfide levels with CTS, detailed in Figure 3. The results are summarized as follows: A significant correlation was found between glucuronate levels and CTS, evidenced by an Odds Ratio (OR) of 0.98 and a 95% Confidence Interval (CI) of 0.97–0.99, achieving statistical significance with an FDR-adjusted *p*-value of 0.002. Moreover, the ratio of AMP to phosphate exhibited a significant association with CTS, with an OR of 0.58 and a 95% CI of 0.45–0.74, and an FDR-adjusted *p*-value of 0.009. Additionally, a notable correlation was observed between cysteinylglycine disulfide levels and CTS, indicated by an OR of 0.85 and a 95% CI of 0.78–0.92, and an FDR-adjusted *p*-value of 0.047. Seventy-four other metabolites (and metabolite ratios) were also identified as having a potential causal relationship with CTS. To corroborate these findings, sensitivity analyses were performed using the MR-Egger and leave-one-out methods, further validating the identified associations, as illustrated in Supplementary Figures S1–S6.

## 4 Discussion

CTS is a common peripheral neuropathy characterized by compression of the median nerve within the carpal tunnel. Various risk factors have been identified, including diabetes (Pourmemari and Shiri, 2016), menopause (Mondelli et al., 2002), hypothyroidism (Shiri, 2014), obesity (Shiri et al., 2015), pregnancy (Padua et al., 2010), and rheumatoid arthritis (Kaya Subaşı et al., 2021). While the relationship between metabolomics, pain, and peripheral neuropathy has been extensively studied (Teckchandani et al., 2021b; Dopkins et al., 2018), its connection with CTS remains unclear. Our study aims to comprehensively investigate the individual causal effects of a wide range of circulating metabolic traits on the risk of developing CTS, thus contributing significantly to the understanding of this condition. This study successfully identified at the SNP level that glucuronic acid, the ratio of AMP to phosphate, and cysteinylglycine disulfide have a significant impact on CTS. These findings offer valuable insights into the metabolic factors contributing to the development and prevention of CTS.

### 4.1 Causal relationship between glucuronate and CTS

Glucuronate is a type of glucuronic acid, which is formed by combining glucose molecules with aldehyde acid (glucuronic acid) molecules (Dowben, 1956). Glucuronate has a variety of physiological roles in the human body, such as detoxification, antioxidant, maintenance of joint lubrication (Kroemer and Klotz, 1992; Zenser et al., 1999; Fabregat et al., 2013; Nash and Prather, 2023). Glucuronic acid has a role in maintaining joint lubrication because of its involvement in the production of hyaluronic acid (Bastow et al., 2008). Hyaluronic acid has good lubricating properties that reduce friction between joints, tendons and other tissues, providing good protection and sliding surfaces (Morgese et al., 2018; Marian et al., 2021). Due to excessive force or inflammation of the carpal joint, etc., the hyaluronic acid in the carpal joint continues to decrease and the pressure in the carpal tunnel increases. Increased pressure in the carpal tunnel is a common mechanism of CTS. Because glucuronic acid-mediated production of hyaluronic acid may be able to reduce the occurrence of CTS. In a systematic review, it was also noted that hyaluronic acid injections significantly relieved pain in CTS and enhanced median nerve function (Urits et al., 2020). Another observational study also suggests that hyaluronic acid injections may have a therapeutic effect in mild or moderate CTS (Su et al., 2021).

### 4.2 Causal relationship between cysteinylglycine disulfide levels and CTS

The results of this MR study suggest a negative association between cysteinylglycine disulfide and CTS. However, there are limited observational findings to confirm this relationship. Prolonged compression of the median nerve in CTS leads to nerve ischemia and hypoxia, which is considered a significant cause of CTS (Sud and Freeland, 2005). The ischemic-hypoxic injury results in oxidative stress in the median nerve, leading to the symptoms of CTS. An observational study showed a strong correlation between CTS and antioxidant, indicating that increased total oxidative stress and oxidative stress indices, along with decreased total antioxidant status, may contribute to fibrosis through disrupted signaling patterns in the tendon sheath and median nerve (Demirkol et al., 2012). Another study revealed that oxidative stress in the subsynovial connective tissue is associated with CTS and its symptoms (Kim et al., 2010). Therefore, antioxidant therapy might be able to reduce the occurrence of CTS. Cysteine glycine disulfide, which is a metabolic breakdown product of cysteine, plays a crucial role as an antioxidant and helps cells combat oxidative stress (Wei et al., 2020). It acts as an important cellular antioxidant, maintaining a balance between the production and scavenging of free radicals to protect neurons from oxidative damage (Gao et al., 2020). Therefore, in the future, cysteine glycine disulfide could be investigated as a potential therapeutic agent for the treatment of CTS.

### 4.3 Causal relationship between AMP to phosphate ratio and CTS

This study shows that AMP to phosphate ratio is negatively correlated with CTS. Previous studies have reported that AMP has a regulatory role in the nervous system, where it affects neurotransmitter release and neuronal excitability, thereby modulating neurotransmission and neural function (Bading, 2017). AMP can activate adenosine monophosphate protein kinase (AMPK) during cellular hypoxia, and AMPK is an important cellular energy sensor and regulator that plays a key role in cellular energy metabolism (Alnaaim et al., 2023). When the cellular energy level decreases, AMPK is activated by AMP, which initiates a series of regulatory mechanisms to increase cellular energy production and decrease energy expenditure, thereby maintaining cellular energy homeostasis (Wang et al., 2023). CTS is caused by hypoxia of the median nerve due to the carpal tunnel squeezing the median nerve. Thus, the activation of AMPK by AMP during median nerve hypoxia may be able to participate in the regulation of the median nerve to achieve its protective effect. Phosphate is an inorganic salt that plays an important role in organisms, participating in energy metabolism, DNA and RNA synthesis, and so on (Gaire and Choi, 2021). If the concentration of phosphate is too high, it can lead to phosphate deposition may lead to carpal tunnel stenosis and CTS (Atreya and Saba, 2022). Therefore, AMP to phosphate ratio may also be useful as a metabolic marker for the study of CTS.

### 4.4 Potential causal relationships between 74 metabolites (ratios) and CTS

In the present study, we also identified 74 metabolites as potential protective or risk factors for carpal tunnel syndrome. Ascorbic acid 2-sulfate, Glycochenodeoxycholate and Histidine to glutamine ratio may be potential protective factors. Analysis by metabolic analysts showed that Ascorbic acid 2-sulfate, a metabolite of vitamin C, has antioxidant and anti-inflammatory effects and helps protect cells from oxidative stress. Glycochenodeoxycholate, a metabolite of bile acids, may maintain bile acid homeostasis, protect hepatic function, and reduce the carpal tunnel syndrome Normal levels of Histidine to glutamine ratio may help maintain amino acid metabolic homeostasis, reduce neuronal dysfunction, and decrease the risk of carpal tunnel syndrome. On the other hand, metabolites such as n-acetylaspartate (NAA) levels, ceramide (d18:1/16:0), and ethylmalonate may be considered as potential risk factors. Changes in levels of n-acetylaspartate (NAA), a metabolite in neurons and glial cells may reflect neuronal damage and correlate with the pathogenesis of carpal tunnel syndrome. Ceramide (d18:1/16:0) is a lipid metabolite, and increases may cause apoptosis and an inflammatory response, exacerbating damage to nerve and muscle tissue and increasing the risk of carpal tunnel syndrome. Abnormal levels of ethylmalonate may lead to metabolic disturbances, increase intracellular oxidative stress and inflammatory responses, affecting the development of carpal tunnel syndrome. In addition, compounds such as X-21470 levels, X-22771 levels, Methyl indole-3-acetate levels, X-23739 levels, and Sucrose levels may be related to the regulation of metabolic pathways in certain organisms, and their physiological significance needs to be further investigated. The mechanisms of action and interrelationships of these metabolites are important for a better understanding of the pathogenesis and treatment of carpal tunnel syndrome.

### 4.5 Strengths and limitations

Our study marks a significant advancement in understanding CTS by being the first to analyze its causal relationship with metabolomics using MR. This approach allowed us to genetically identify glucuronate, the ratio of AMP to phosphate, and cysteinylglycine disulfide levels as a protective factor against CTS. The strength of MR lies in its ability to minimize confounding factors and reduce reverse causation, as gene variants are randomly assigned at conception and remain independent of subsequent lifestyle and environmental influences. To ensure the robustness of our findings, we utilized metabolic biomarker GWAS data from the Genome-Wide Association Studies (GWAS) Catalog database and genetic variation data for CTS, the FinnGen database. These carefully selected instrumental variables enhance the credibility of our MR analysis. We also employed MR-Egger regression and MR-PRESSO to address potential issues like horizontal pleiotropy and heterogeneity.

However, our study is not without limitations. The limited number of SNPs, resulting in the reduced stability of this study’s findings regarding the AMP to phosphate ratio and cysteinylglycine disulfide levels, may be attributed to the relatively small sample size, which covered only 8,299 of the 1,400 metabolites. To enhance the reliability of the causal relationship in future research, it is advisable to expand the sample size beyond 1,400 metabolites. Although SNPs were used in this study to correlate with metabolite levels, other factors also influence metabolite levels, such as environmental influences, dietary habits, and lifestyle choices also play a significant role in determining metabolite concentrations. So it is possible that other factors may further influence CTS. Therefore, future causal relationships between metabolites and CTS will require multicenter clinical studies to further validate their relationship.

## 5 Conclusion

In summary, the results of this study suggest that the identified glucuronate, the ratio of AMP to phosphate, and cysteinylglycine disulfide levels can be considered as metabolic biomarkers for CTS screening and prevention in future clinical practice, as well as candidate molecules for future mechanism exploration and drug target selection.

## Data Availability

The original contributions presented in the study are included in the article/Supplementary Material, further inquiries can be directed to the corresponding author.
