# Supplementary figures and images for: Causal relationship between plasma metabolites and carpal tunnel syndrome risk: evidence from a mendelian randomization study
Source: Front Mol Biosci. 2024 Oct 3;11:1431329. doi: 10.3389/fmolb.2024.1431329 (PMC11484071; doi:10.3389/fmolb.2024.1431329)

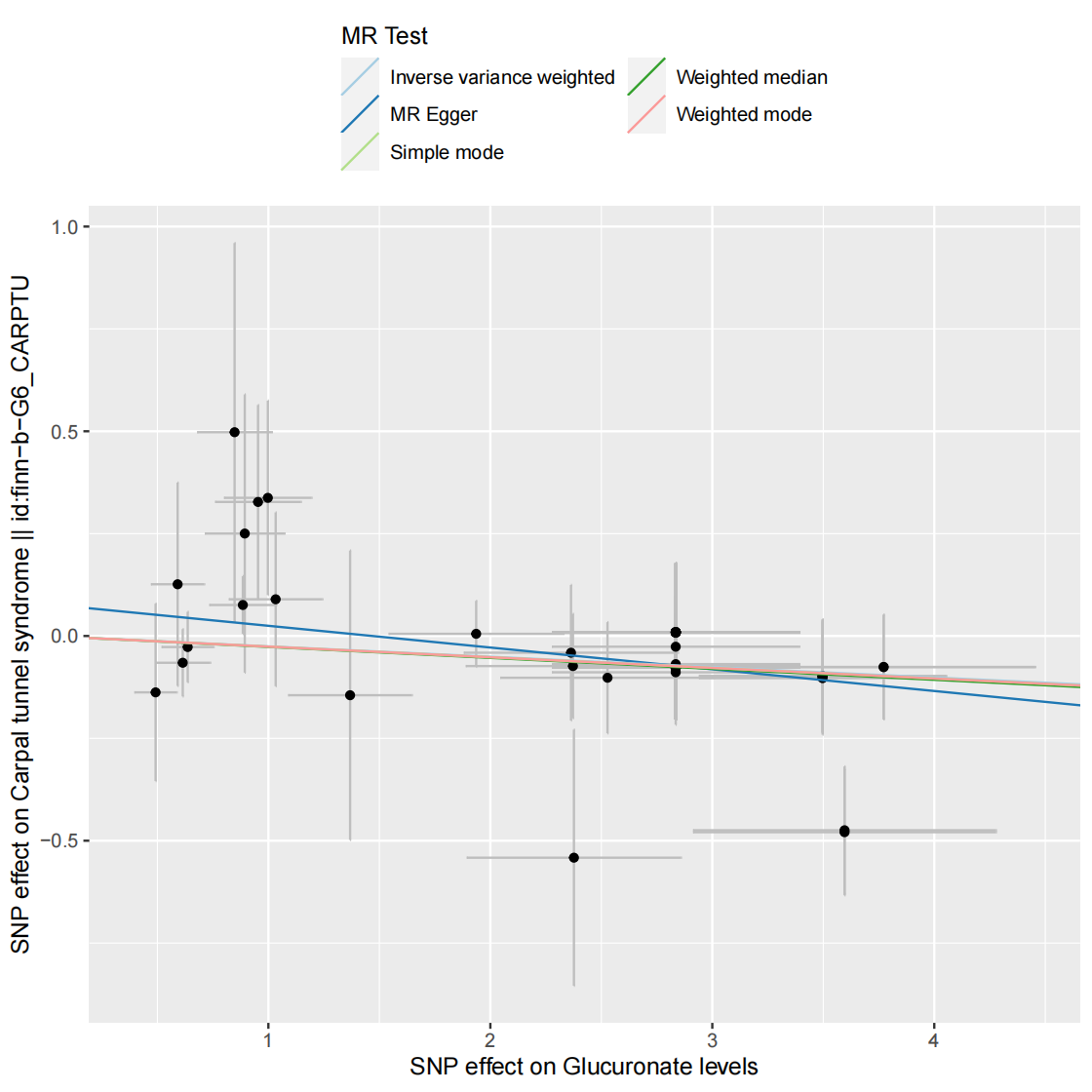

Supplement: Supplementary file 3 [file Image6.TIF]

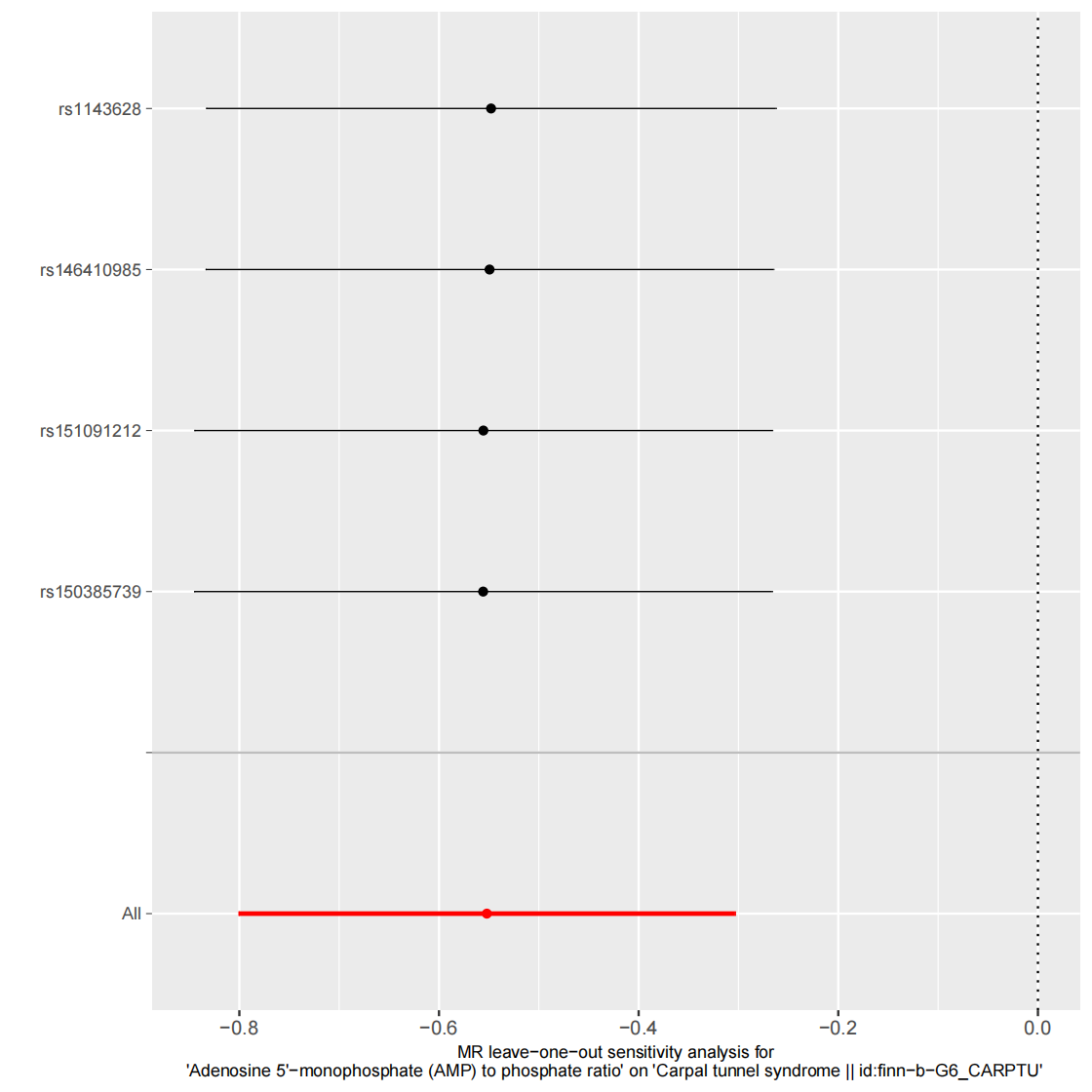

Supplement: Supplementary file 4 [file Image3.TIF]

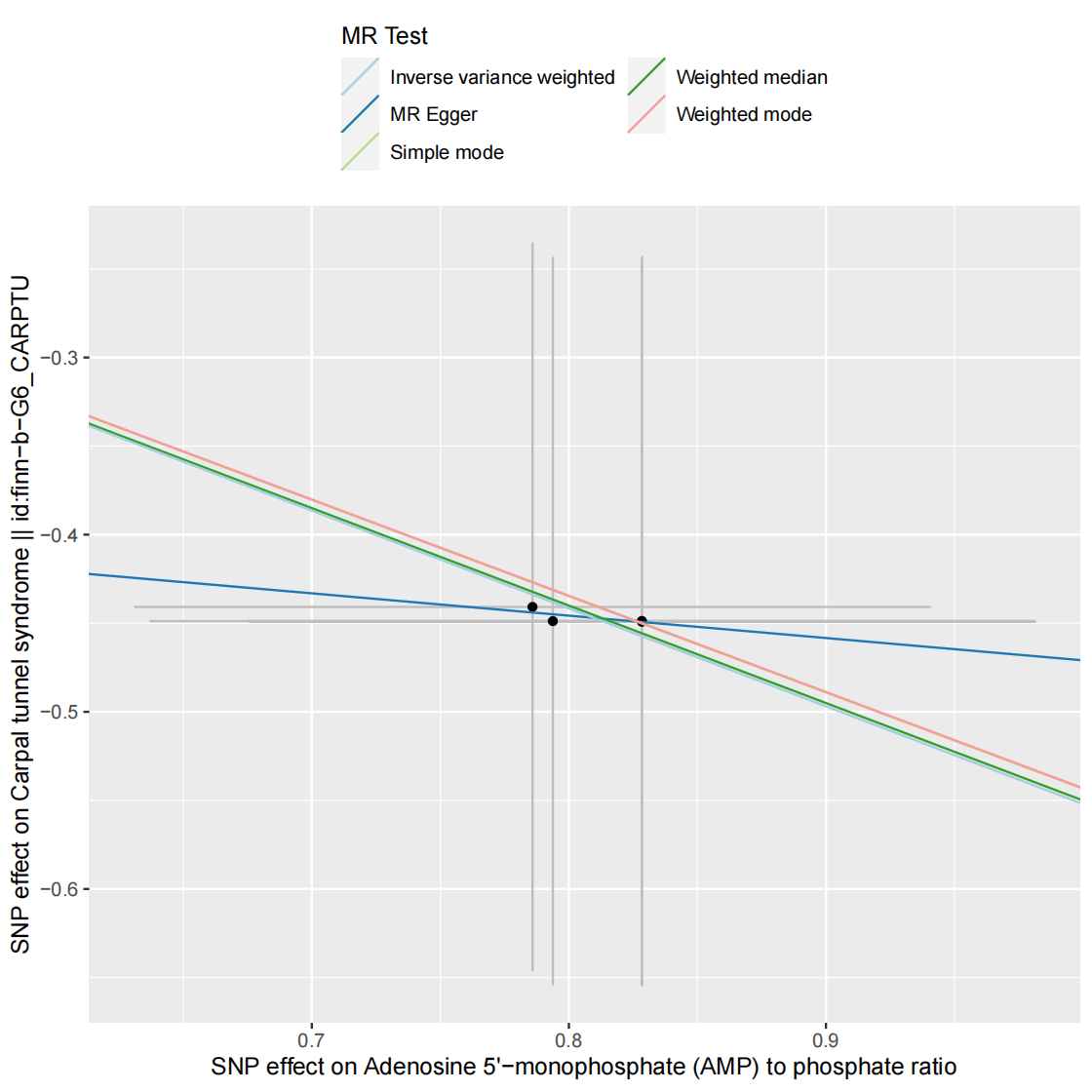

Supplement: Supplementary file 5 [file Image4.TIF]

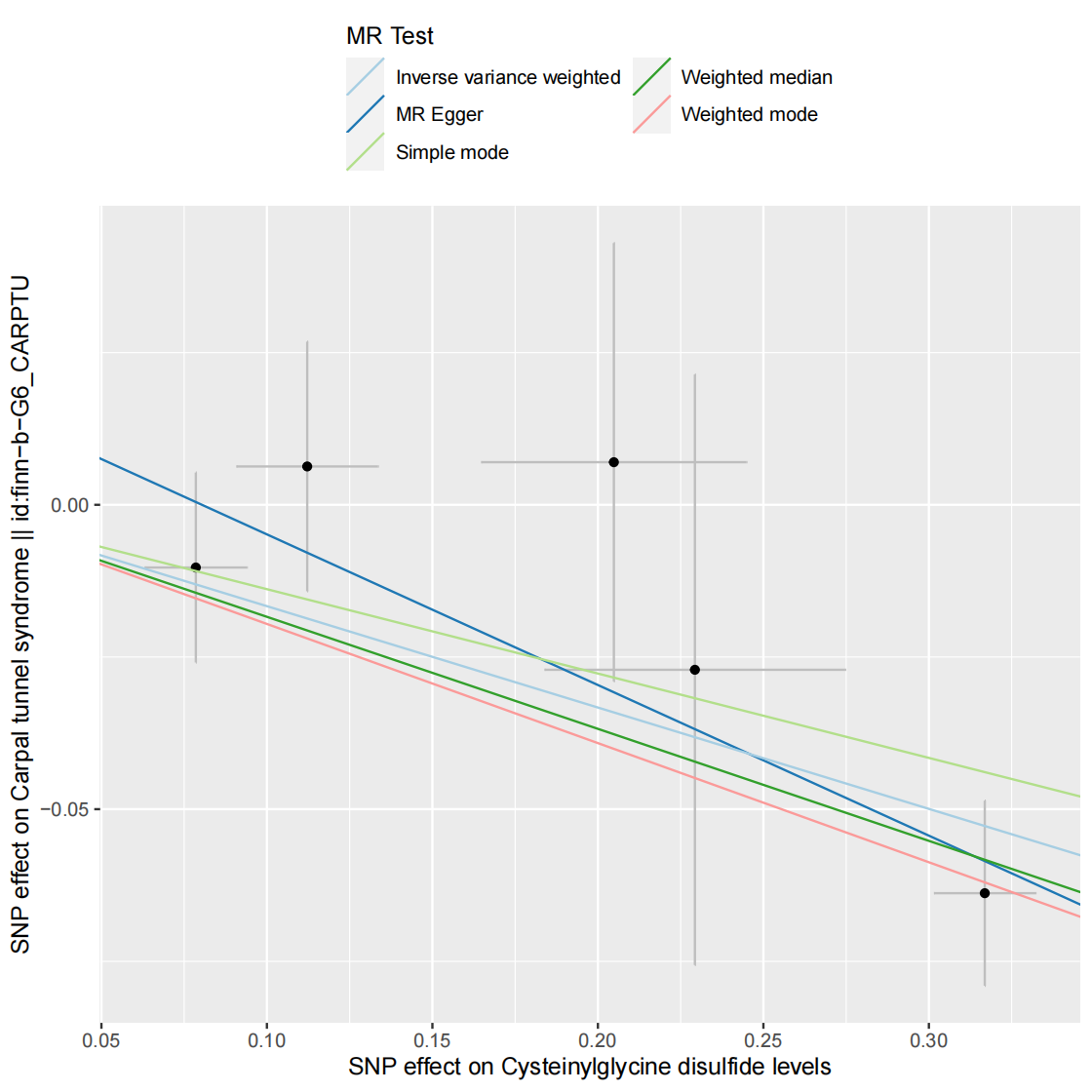

Supplement: Supplementary file 6 [file Image2.TIF]

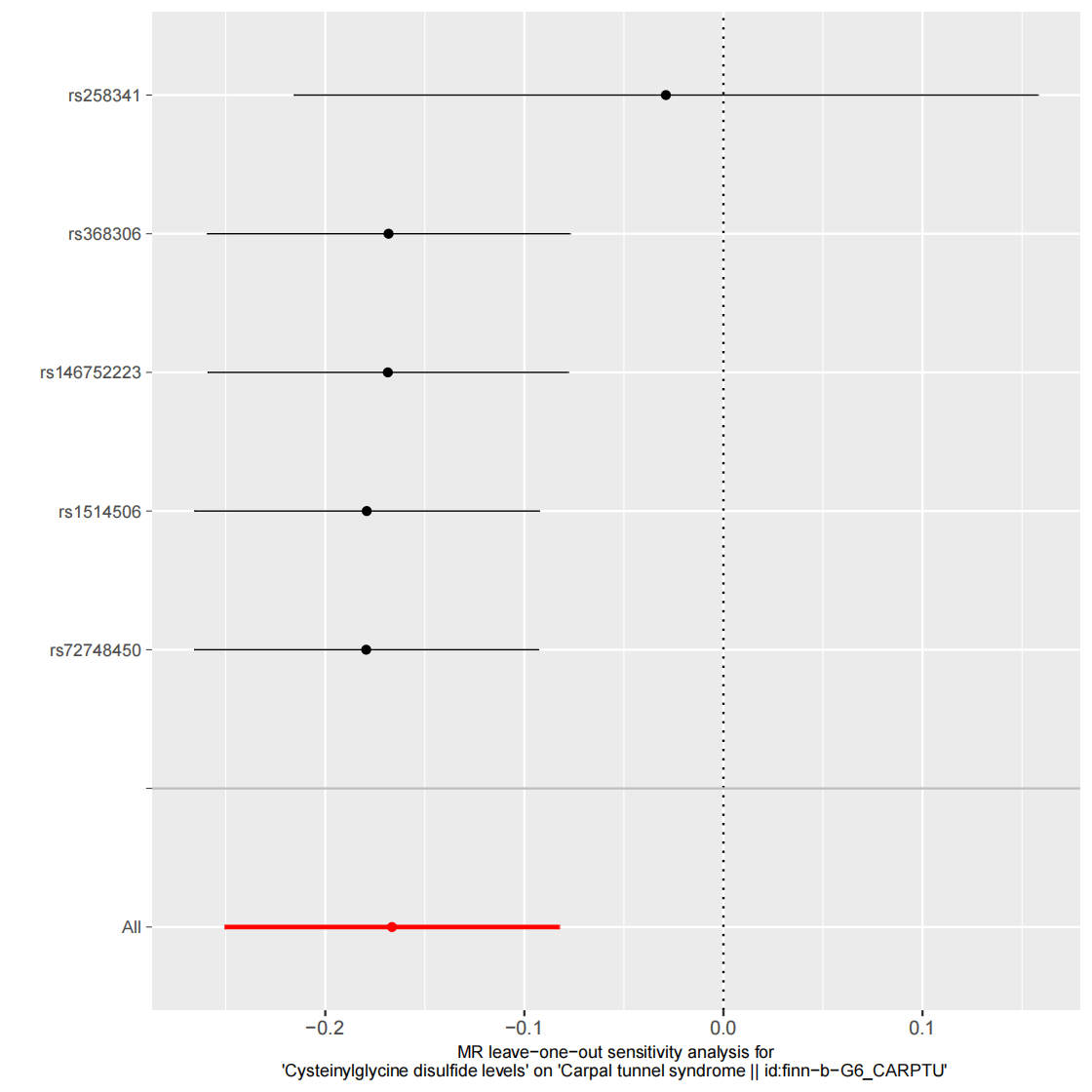

Supplement: Supplementary file 7 [file Image1.TIF]

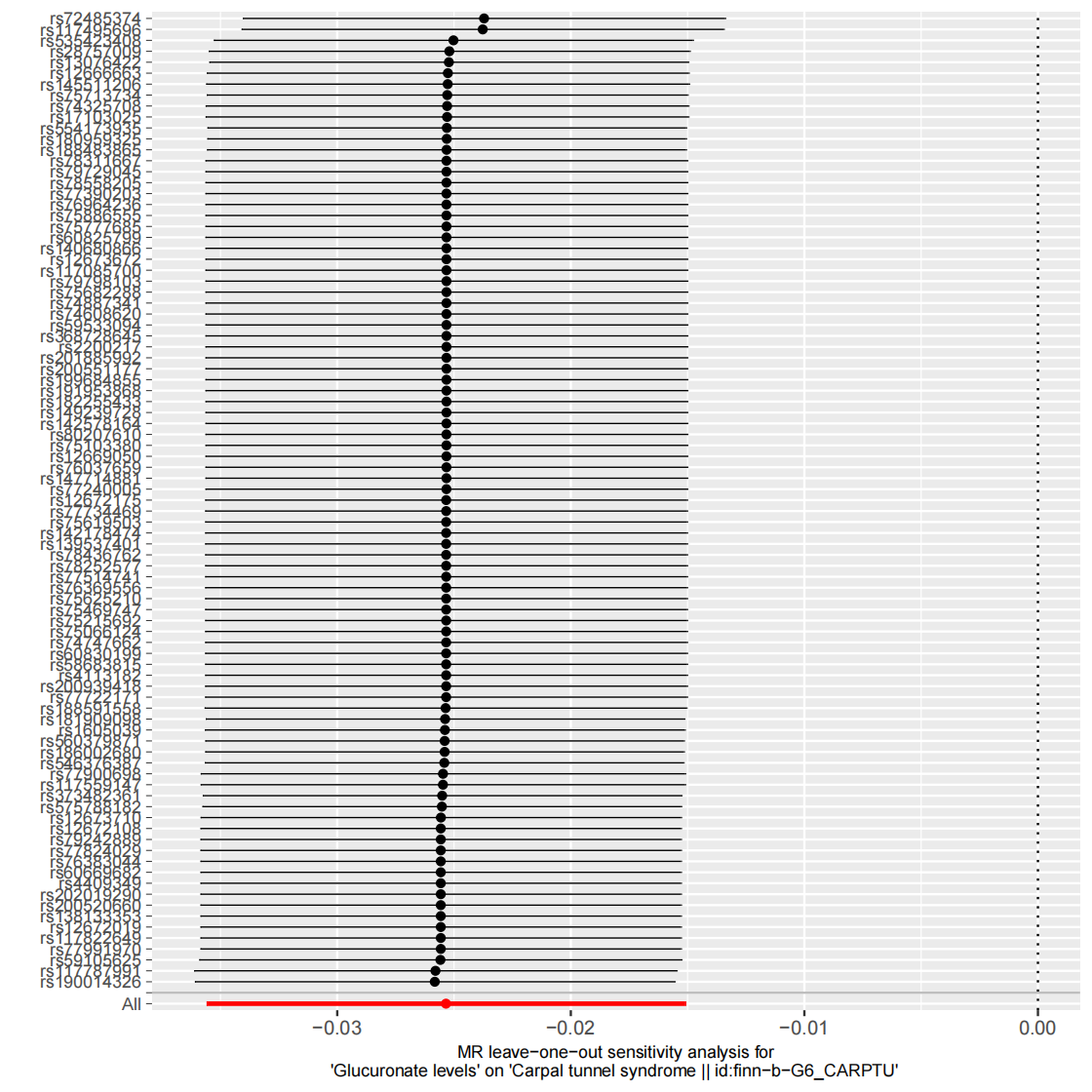

Supplement: Supplementary file 12 [file Image5.TIF]
