# Supplementary material for: Causal relationship between plasma metabolites and carpal tunnel syndrome risk: evidence from a mendelian randomization study
Source: Front Mol Biosci. 2024 Oct 3;11:1431329. doi: 10.3389/fmolb.2024.1431329 (PMC11484071; doi:10.3389/fmolb.2024.1431329)
Supplement: Supplementary file 9 [file DataSheet1.PDF]

```
library(TwoSampleMR)
library(openxlsx)
library(data.table)
ALM_full_outcome<-readRDS("./Carpal_tunnel_syndrome_R10_full_outcome.rds")
lf <-list.files(pattern = ".tsv.gz$")
for(j in 1:1400){
  f<-fread(lf[j])
  f<-subset(f,f$p_value<1e-06)
  f<-data.frame(f)
  a <- format_data(
    f,
    type = "exposure",
    snps = NULL,
    header = TRUE,
    phenotype_col = lf[j],
    snp_col = "rsid",
    beta_col = "beta",
    se_col = "standard_error",
    effect_allele_col = "effect_allele",
    other_allele_col = "other_allele",
    pval_col = "p_value",
    chr_col = "chromosome",
    samplesize_col = "n",
    eaf_col = "effect_allele_frequency",
    pos_col = "base_pair_location"
  )
  a<-clump_data(
    a,
    clump_kb = 500,
    clump_r2 = 0.01,
    clump_p1 = 1e-06,
    clump_p2 = 1e-06,
    pop = "EUR",
    bfile = NULL,
    plink_bin = NULL
  )
  a$id.exposure<-c[j]
  mydata <- harmonise_data(
    exposure_dat=a,
    outcome_dat=ALM_full_outcome,
    action= 2)
  het=mr_heterogeneity(mydata)
  het_all<-rbind(het_all,het)
  pleio=mr_pleiotropy_test(mydata)
```

```
    pleio_all<-rbind(pleio_all,pleio)
    res_mi=generate_odds_ratios(mr(mydata,method_list=c("mr_ivw","mr_wald_ratio")))
    res_mi_all<-rbind(res_mi_all,res_mi)
}
writexl::write_xlsx(x = res_mi_all,"res_mi_all2xlsx")
writexl::write_xlsx(x = pleio_all,"pleio_all2.xlsx")
writexl::write_xlsx(x = het_all,"het_all2.xlsx")
```
